# Cysticercus Cellulosæ

**Published:** 1879-12

**Authors:** 


### CYSTICERCUS CELLULOSÆ.

Dr. C. S. Turnbull relates (Transactions of the Medical Society of Pennsylvania, Vol. XII, 1879) a case of cysticercus within the human eye, and gives with it a brief account of the parasite and of other cases of the same sort on record.

The patient was a merchant, aged fifty-five years, who had received an injury to the eye, subsequently affected, in childhood. Sight became gradually dim in this organ from opacity of the lens. Five years since, without any explainable cause, the cataractous eye, which had given him no special trouble, became the seat of paroxysmal pains, recurring with a frequency and regularity suggestive of malarial influence. A peculiarity of diagnostic signification in connection with these pains lay in a gradually increasing intensity characterizing them. The cause was evidently of a growing nature, and as a culmination of this intense pain the patient became conscious of blows struck on the inside of the eye, the sensation being described as "hammer strokes." The pains were accompanied with marked vascular engorgement, the conjunctivæ would become congested, and lachrymation was usually profuse. The paroxysms usually came on about bi-weekly.

After treating the granular condition of the lids, which was present, and various methods had been tried to cure the "neuralgia," supposed to be the morbid condition to be dealt with, the pupil was fully dilated with atropia. Now the cause of all trouble became visible. A balloon-shaped body was seen floating in the aqueous humor. The parasite was living and showed active movements.

"To illustrate the movement of the animal, reference may be made to a balloon. A cysticercus is, in form, a miniature balloon, the head of the parasite, which is the pendant part, bears close likeness, when extended, to the basket. To imagine a floating and swaying balloon suddenly drawing into its interior, and as suddenly throwing out its basket, is to secure the fullest notion of the action of the hydatid." The parasite having fixed itself to the centre of the capsule of the crystalline lens, it was removed by the performance of Graefe's method for extraction of the lens. It lived for some time afterwards in a warm saline solution. The description and operation are by Dr. J. E. Garretson.

This parasite is the larval form of what is known as the Tænia solium, or tape-worm. The person infected may derive the mature eggs of the tænia from his own intestines or from the tape-worm of some one else. The detached and expelled joints of a tape-worm may be taken into the mouth and swallowed, or the mature egg-containing segments may, by vomiting, be regurgitated into the stomach. In the second way by food as through the use of cooking utensils. Every person affected with tape-worm not only carries danger to himself with him, but is constantly threatening the health of his neighbors. The eggs of the tape-worm, when swallowed, develop into cysticerci, while the cysticerci, while taken into the digestive tract, develop into tape-worms.

A practical point is quoted from Cobbold: A reference to the handling of fresh tape-worms, their eggs might get under the finger nails or upon the clothes, and from thence to the digestive tract. Also the danger of eating salads which may have been manured with night soil containing, perhaps, myriads of tape-worm eggs. A cooking temperature of 140° F. is sufficient to kill cysticerci of all kinds.—*St. Louis Clinical Record.*

